# Preschoolers' Empathy Profiles and Their Social Adjustment

**DOI:** 10.3389/fpsyg.2021.782500

**Published:** 2021-12-10

**Authors:** Poline Simon, Nathalie Nader-Grosbois

**Affiliations:** Chair Baron Frère in Special Education, Psychological Sciences Research Institute, Université Catholique de Louvain, Louvain-La-Neuve, Belgium

**Keywords:** personality, affective empathy, cognitive empathy, social adjustment, preschoolers

## Abstract

Preschoolers face new challenges in their social life: the development of social and emotional abilities in order to have positive relationships with peers and adults. Empathy, the ability to share and understand the emotions of others, contributes to this socio-emotional adjustment. This exploratory study examines mothers and fathers' perceptions of their child's empathy and individual factors, such as age, gender, and personality, which are related to cognitive and affective empathy in 63 typically developing preschoolers. Links between children's individual characteristics (empathy and personality) and their social adjustment on the one hand and risk of developing internalized vs. externalized behaviors on the other were also investigated. Parents completed four questionnaires about their child's empathy, personality, and social (mal)adjustment. The results showed that mothers and fathers perceived their children's cognitive and affective empathy, attention to others' feelings, and social actions (such as helping), in the same way, except for emotion contagion. Gender differences appeared specifically for some components of empathy: girls were said to pay more attention to others' emotions while boys had better cognitive empathy. Moreover, children's empathy as perceived by mothers or fathers was positively linked with their age, and with personality factors (extraversion, emotional stability, agreeableness, and openness to experience). Cognitive empathy and personality were found to be partly related to higher social skills and lower externalized and internalized behaviors. The results nuanced specific links between cognitive and affective empathy and social adjustment as well as behavior problems at preschool age. These results may have some implications for future research and prevention in childhood.

## Introduction

Preschoolers face new challenges when they enter kindergarten. In this social environment, their social interactions multiply and they experience or witness various emotional situations with peers and adults, in school environment. In order to experience positive social relationships with other children and to behave in a socially appropriate way, they must regulate their emotions, be open to others' perspective, cooperate, and respect social conventions or rules, depending on the context. In addition, preschoolers need to learn about the sharing of positive and negative emotions felt by others or by themselves. They themselves may experience difficult social situations, conflict, aggressive behavior, isolation, or distress, or they may see other children doing so. Preschoolers may have difficulties in managing their emotions and social behaviors, in responding adequately in situations of this kind or in expressing empathy toward another child and giving help. At this age, the most significant mental health risks are externalizing problems (e.g., verbal and physical aggression, opposition, irritability, and bullying) and internalizing problems (e.g., anxious or depressive disorders, isolation), which need to be identified and addressed in targeted interventions in order to prevent them during childhood. The challenge for parents and teachers is therefore to observe, to understand strengths and weaknesses in children's socioemotional profiles, and to identify any victims, bullies, or witnesses in critical social situations. To better understand socio-emotional abilities and take effective steps in response to these early signs of bullying/aggression or internalizing behaviors in young children as soon as possible, it is essential to assess and boost children's socioemotional skills and to promote prosocial and social behaviors. Relevant theoretical models and empirical research must guide assessment and evidence-based interventions for preschoolers, taking into account their social information processing (SIP, Crick and Dodge, 1994), their understanding of affective and cognitive mental states (Theory of Mind, ToM, Flavell, 1999), their emotion regulation in social interactions (Shields and Cicchetti, 1997), and their empathy abilities (Hoffman, 2000). Assessment and intervention should also reflect the way in which these skills are linked and contribute to social adjustment or maladjustment (Denham et al., 2003; Yeates et al., 2007; Nader-Grosbois and Thirion-Marissiaux, 2011). Numerous studies have investigated bi-directional and predictive links at preschool age, between emotion regulation or social adjustment, and SIP (Yeates et al., 2007; Barisnikov and Hippolyte, 2011), or ToM (e.g., Deneault et al., 2011; Deneault and Ricard, 2013).

### Empathy Development and Components

Empathy is defined as an emotional response that arises from understanding the emotions of others (Eisenberg et al., 2006, p.647). In the developmental model of empathy of Hoffman (2000), five stages are explained, from early signs of empathy in babies to true empathy, which carries on developing until adulthood. Hoffman (2000) describes the different empathic behaviors and reactions of children as they grow up when confronted with the distress of others. In the first stage, called *newborn reactive cry*, and up to the age of 6 months, babies cry when they hear another baby crying. At the end of the first year of life, in the *egocentric empathic distress* stage, infants always react to others' distress, but in a less intense way. They confuse their distress with that of others, but begin to adopt some behaviors that soothe their own pain. During the *quasi-egocentric empathic distress* stage, at the beginning of the second year of life, toddlers understand that another person is in distress, feel it, and attempt to comfort or to help the person by displaying some behaviors which help to soothe their own distress but are not adapted to overcoming the other person's distress (e.g., giving their own security blanket to another child). The *veridical empathic distress* stage develops from the end of the second year, by which time children understand what people feel and that their own internal state is distinct from others' internal state, and are able to take others' perspective and respond to the perceived needs of the distressed person in a prosocial way. Between 5 and 8 years old, in the *empathic distress beyond the situation* stage, children can understand that people can feel emotions in general contexts of life and not only in the situation experienced at that time. Few studies have examined empathy and prosocial behaviors in preschoolers using validated and adapted measures based on developmental theoretical conceptions such as adult-reported questionnaires (Belacchi and Farina, 2012), performance-based measures or observational designs (Bensalah et al., 2016). For example, to apprehend developmental changes at early and preschool age, Rieffe et al. (2010) have created a questionnaire that includes three subscales, inspired by the first three stages of this developmental model. The first subscale concerns *emotion contagion*, which refers to automatic imitation and synchronization of other person's expressions, vocalizations, and behaviors (Hatfield et al., 1993). The second subscale, called *attention to others' feelings*, refers to the child's capacity to be aware of others' emotions. The third subscale covers the *prosocial actions* provided by the child to respond to others' emotions (Rieffe et al., 2010).

Since the 1980s, researchers in developmental psychology and developmental neuropsychology have postulated distinct components in empathy and investigated their characteristics in typical and atypically developing populations. “Affective or emotional empathy” refers to the capacity to share in other people's emotional state, while “cognitive empathy” corresponds to the ability to understand others' emotions (Decety et al., 2015). Most of models conceive empathy as a bi-dimensional construct but some authors integrate other components in their model, such as motor empathy (Blair, 2005) and emotion regulation (Decety and Jackson, 2006). Recently, a “behavioral” component of empathy has been introduced into some conceptual models to describe behaviors that arise from affective and cognitive empathy. These behaviors may or may not be socially appropriate (Rieffe et al., 2010; Reid et al., 2013; Bensalah et al., 2016). However, in empirical studies, prosocial behaviors are often perceived as observable consequences of empathy rather than as a distinct component of empathy. In this view, a majority of studies targeted affective and cognitive empathy, or only one of these two components.

Some authors claim that children's affective empathy remains stable over time in childhood (Roberts and Strayer, 1996; Schwenck et al., 2014; Bensalah et al., 2016), while others suggest that it is subject to development (Rieffe et al., 2010). Other studies have highlighted improvements in cognitive empathy as children grow older (McDonald and Messinger, 2011; Davidov et al., 2013; Schwenck et al., 2014). At preschool and school age, it has been all too common for studies to treat empathy as a unidimensional construct and not differentiate between affective and cognitive components, despite the existence of measures capable of recording both components. For example, questionnaires such as the Griffith Empathy Measure (GEM, Dadds et al., 2008) can be used to create profiles based on specific “affective” and “cognitive” scores rather than just a global empathy score.

### Empathy, Prosocial and Social Behaviors, Externalizing, and Internalizing Problems

Research has shown that empathy plays a role in protecting social abilities and positive relationships (Mayberry and Espelage, 2007; Girard et al., 2014) and promotes cooperation and group cohesion (Zahn-Waxler et al., 1992; Jolliffe and Farrington, 2006b). However, the positive link between empathy and prosocial behaviors has been mainly studied in school-age children (e.g., Girard et al., 2014; Murakami et al., 2014; Deschamps et al., 2015; Taylor and Glen, 2020) and specifically in adolescents (e.g., LeSure-Lester, 2000; Wang et al., 2019), but to a less extent at preschool age (e.g., Roberts and Strayer, 1996; Williams et al., 2014). Most of these studies have treated empathy as a unidimensional construct, or have only taken one of the two components of empathy into account. For example, Williams et al. (2014) emphasized that empathic 3- to 6-year-old children demonstrated more prosocial behaviors, sharing more with others and withholding fewer benefits for themselves. Moreover, this study indicated that prosocial behaviors were more motivated by empathic concern for others' emotions than by personal distress. Roberts and Strayer (1996) found the same results on a sample with a larger age range, from 5- to 13-years old, divided into three groups (5, 9, and 13 years old). This research reflected results obtained by other authors with young (Zahn-Waxler et al., 1992), preschool (Strayer and Roberts, 2004; Eisenberg et al., 2010; Zava et al., 2021), and school-age children (Warden and Mackinnon, 2003; Deschamps et al., 2015), and adolescents (LeSure-Lester, 2000; Wang et al., 2019). According to Wang et al. (2019), children and adolescents with high level of empathy are more accepted by their peers due to their prosocial behaviors (but also their low level of aggression). Two recent studies have investigated this relation considering the affective and cognitive components of empathy. For example, Belacchi and Farina (2012) reported that preschoolers aged between 3 and 6 years who are perceived by their teachers as defenders and mediators (prosocial role) have better affective empathy, although cognitive empathy is not taken into account. Cavojová (2012) obtained the same conclusion with adolescents: those who were perceived by their peers as prosocial display higher affective empathy but also better cognitive empathy. However, these two studies took place in school environment and did not consider the parent's point of view regarding social situations of children's daily life in other contexts.

Conversely, weaker empathy is thought to entail a risk of inappropriate social behaviors, displayed through aggressivity and conflict, for example (Dahmen et al., 2004). Most studies have emphasized that empathy is negatively related to externalizing problems at an early age (Noten et al., 2020), preschool age (Strayer and Roberts, 2004; Ekerim-Akbulut et al., 2020), and school age (Deschamps et al., 2015, 2018; Malcolm-Smith et al., 2015), and in adolescence (LeSure-Lester, 2000; Lovett and Sheffield, 2007; Pouw et al., 2013). Feeling the same emotion as others discourages children from acting inappropriately, for example by hitting or mocking them (Hastings et al., 2000). Conversely, a low level of empathy is positively associated with aggressive or antisocial behaviors in preschoolers (Belacchi and Farina, 2012), schoolers (Deschamps et al., 2018), and adolescents (Jolliffe and Farrington, 2007). It prevents them from understanding and responding appropriately to the emotional states of others or from controlling their own states (Vachon et al., 2014). As for the relation between empathy and prosocial behaviors, a few studies have analyzed the contribution of affective and cognitive empathy to antisocial behaviors. Belacchi and Farina (2012) highlighted that hostile behavior is negatively predicted by affective and cognitive empathy. In other words, preschoolers perceived as bullies by their teachers have low levels of affective and cognitive empathy. These results partially corroborate those of the study of Jolliffe and Farrington (2006b), in which adolescents' affective empathy (but not their cognitive empathy) was found to be negatively related to bullying. The authors explained that these results are consistent with the empathy profiles of children and adolescents with externalized behavioral problems, who seem to have a deficit in affective empathy but not in cognitive empathy. Sufficient cognitive empathy skills allow bullies to understand others' emotions, to know exactly what to do to hurt others without feeling any emotions (Sutton et al., 1999).

Although most studies confirm the negative link between empathic skills and externalizing behaviors, several authors have reached more nuanced conclusions. In their meta-analysis, Miller and Eisenberg (1988) and Lovett and Sheffield (2007) posit that the relation between empathy and aggressivity is more robust when children are older. By contrast, Hastings et al. (2000) demonstrated that a high level of aggressivity can coexist with a good level of empathy in children aged between 4 and 10 years.

Concerning internalizing behaviors, the literature shows that extremely high levels of empathy lead to higher levels of internalizing behaviors (Tibi-Elhanany, 2011; Pechorro et al., 2015), but Tone and Tully (2014) state that this relation exists when other factors are combined. For example, Tully and Donohue (2017) compared the link between affective and cognitive empathy and internalizing behaviors in children of depressed and non-depressed mothers. The results showed that children of depressed mothers presented a higher level of internalizing behaviors when they expressed higher levels of affective and cognitive empathy; comparison with children of non-depressed mothers suggested that a mother's depression plays a role in the development of children's internalizing behaviors relative to their empathy. Conversely, children of non-depressed mothers who had a higher level of affective empathy with regard to happiness did not have internalizing difficulties. Raine and Chen (2018) obtained the same relation for cognitive empathy: children with better cognitive empathy skills are less withdrawn. However, these results should be considered with caution because studies of the link between empathy and internalizing behaviors are very scarce at preschool age (Raine and Chen, 2018).

### Empathy Development Related to Different Factors

It is well-known that empathy is influenced by genetic factors (Knafo et al., 2008), individual factors (such as age, gender, or personality), and factors related to family environment. However, abilities in other domains are also involved in empathy development, such as attachment, language, and cognitive skills (McDonald and Messinger, 2011; Davidov et al., 2013; Stern and Cassidy, 2018).

Although the potential gender difference in empathy has been widely studied, the results are controversial. Considering empathy as a unidimensional construct, most of studies have emphasized that girls are more empathic than boys (Zahn-Waxler et al., 1992; de Wied et al., 2007; Auyeung et al., 2009; Lucas-Molina et al., 2018). The authors of these studies argue that children learn at an early stage the roles assigned to their gender, which is why girls are more concerned about others' emotions than boys, in accordance with their caregiver role (Strauss, 2004). However, some authors have nuanced these conclusions, studying gender influence on affective and cognitive components separately. Some of these studies found that girls have better affective and cognitive empathy than boys (Strayer and Roberts, 2004; Gini et al., 2007; Belacchi and Farina, 2012), while others failed to show any gender differences in either component (Bensalah et al., 2016). A further group of studies have arrived at more balanced results, finding, for example, that at an early age, girls have better affective empathy whereas boys have better cognitive empathy (Volbrecht et al., 2007). Reid et al. (2013) found that preschool girls have better cognitive empathy than boys but that their level of affective empathy is equal, while Schwenck et al. (2014) observed the reverse. Fabes and Eisenberg (1998) argued that the disparity of results could be explained by the different methodologies employed to measure empathy profiles and analyze the gender effect.

Although some authors consider empathy to be a personality trait (Davis, 1980, 1983; Hoffman, 1982; Jolliffe and Farrington, 2006a), others have explored how temperament in young children or personality factors could vary the development of empathy. Concerning preschoolers, a few studies have considered inhibited the link between temperament or shyness and empathy, reaching inconsistent results (Findlay et al., 2006; Cornell and Frick, 2007; Zava et al., 2021): children with an inhibited temperament, who are more shy and fearful with unknown people or situations, are characterized as more empathic by parents (Cornell and Frick, 2007) but these findings contrast with those of Findlay et al. (2006), who indicated that shy preschoolers presented more difficulties in empathy. Liew et al. (2011) reported that fearful children are more affected by personal distress, which forces them to disengage from social (or non-social) activities. However, Zava et al. (2021) did not observe any significant results concerning the link between empathy and inhibited temperament. Beyond personality factors, some studies have explored specific patterns of empathy in children who exhibit psychopathic traits, characterized by antisocial behaviors, low emotionality, and callousness (Hare, 1995). For example, Dadds et al. (2009) reported that children between the age of 3 and 13 with psychopathic traits exhibited deficits in affective empathy (boys only) that increased with age. Concerning cognitive empathy, deficits increased over time in girls while boys were able to overcome it in adolescence. These results corroborated with those of Jones et al. (2010) for affective empathy and partially for cognitive empathy in adolescence. Indeed, the group of boys with psychopathic traits was found to have similar cognitive empathy skills to the control group. Even if some authors studied specific personality factors or disorders in empathy development, no study examined the link between affective and cognitive empathy and the five-factor personality model at preschool age.

Among predictors of empathy, social adjustment or prosocial behaviors, several studies examined personality factors. In their meta-analysis, Silke et al. (2018) highlighted findings obtained in adolescence: empathy (only cognitive empathy) or prosocial behaviors are positively related to extraversion, openness, conscientiousness, and agreeableness (Jolliffe and Farrington, 2006a; Caprara et al., 2010; Tariq and Naqvi, 2020). Concerning neuroticism, Jolliffe and Farrington (2006a) have shown that this personality factor, in girls, was positively related to affective empathy, but not to cognitive empathy. Conversely, prosocial behaviors seem to be negatively linked with neuroticism (Tariq and Naqvi, 2020), while internalizing behaviors are positively related to neuroticism and negatively to extraversion (Slobodskaya and Akhmetova, 2010; Delgado et al., 2018) and externalizing problems are linked to a less agreeable, conscientious, open, and emotionally stable personality (Meunier et al., 2011).

In terms of developmental factors that have been studied, some studies have argued that progression in empathy is linked to the simultaneous development of executive functions (Davidov et al., 2013) or language abilities (McDonald and Messinger, 2011). Other studies focusing on attachment have demonstrated that secure children are more empathic than insecure children (Stern and Cassidy, 2018). Quality of parent–child relationship and parenting style have also been emphasized as protective factors in the development of empathy. Parents using consistent rules, inductive reasoning, warmth, parental sensitivity, and responsiveness and having high expectations have children with better empathic skills (Miller et al., 1989; Kiang et al., 2004; Vinik et al., 2011; Wagers and Kiel, 2019). Moreover, some parental emotional socialization strategies, such as conversations about emotions with the child, or parental expression of emotions, help him or her to develop empathy skills (Brown and Dunn, 1991; Valiente et al., 2004; Taylor et al., 2013). According to Jambon et al. (2019), it seems that siblings play also a role in the development of empathic skills. Indeed, older brothers and sisters pay attention to and are more concerned about younger children, which amplifies their empathy (Jambon et al., 2019).

### Objective of the Study

The literature highlights that, for both affective and cognitive empathy, the progression from emotion contagion to attention to others' feelings and prosocial actions in empathy development could help to improve our understanding of empathy profiles in preschoolers. The associations of the different components of empathy, depending on age, gender, personality factors, needs to be explored in more detail, and research is needed to examine how social adjustment or maladjustment is promoted or impeded by these factors and by empathy. The present exploratory study aimed to examine (1) how mothers and fathers perceive their child's affective and cognitive empathy, (2) whether their perceptions of empathy vary depending on children's individual factors, including gender, age, and personality, and (3) how social competences or internalizing and externalizing behaviors are linked and predicted by children's cognitive and affective empathy and personality. For each objective, hypotheses were formed:

- In the hypothesis 1, it was expected that parents, due to their co-parenting and shared educational values, would be found to perceive their children's empathy in a similar manner.
- In the hypothesis 2, given the controversial findings about similarities vs. differences in affective and cognitive empathy depending gender, we postulated that gender differences could appear either in affective empathy or in cognitive empathy in preschoolers. Moreover, affective and cognitive empathy were expected to be positively related to age and personality factors and in particular emotional stability, agreeableness, extraversion, openness to experience.
- In the hypothesis 3, according to the literature it was expected that social competences are promoted by higher affective and cognitive empathy and personality factors (emotional stability, agreeableness, extraversion, openness to experience), and that internalizing and externalizing problems are negatively associated to these same variables.

## Methods

### Participants

The participants were 63 typically developing children (36 girls and 27 boys) and their parents (63 mothers and 42 fathers) from the French-speaking area of Belgium. The children were aged from 36 to 79 months (*M* = 54.62; *SD* = 11.174). As inclusion criteria, children had to be between 3 and 6 years old, be in ordinary preschool education and speak French. Children with any deficiency or clinical difficulties (delay, behavior disorders, or clinical diagnoses) were excluded.

Concerning the family's socioeconomic status and sociocultural level, two indicators were collected through a short questionnaire: parents' education level and family income. For education level, the majority of mothers had a high school certificate (20.3%), a bachelor's degree (20.3%), or a master's degree (35.9%). Six mothers had a Ph.D. (10.9%). The majority of fathers had a high school certificate or a master's degree (38 and 35.7% of the sample respectively). Of the remaining fathers, six had a bachelor's degree (14.2%) and one had a Ph.D. (2.3%). This information was missing for one mother and one father. In terms of family income, parents indicated the range into which their monthly salaries and benefits fell on a 13-point scale from 0 to 500 to more than 6,000 euros. The mean income reported by parents was 7.95 points, corresponding to the range 3,000–3,500 euros.

### Measures

Two different empathy questionnaires were used in order to take account of the developmental aspects of empathy described by Hoffman (2000) and the cognitive and affective components of empathy. These were the Empathy Questionnaire (EmQue, Rieffe et al., 2010), inspired by Hoffman's (2000) developmental stages, and the GEM (Dadds et al., 2008), giving a score for each component, both of which were completed by parents. To assess personality and social adjustment, parents completed the Bipolar Rating Scales, based on the Five-Factor Model (EBMCF, Roskam et al., 2000), and the Social Competences and Behavior Evaluation Scale (SCBE, LaFrenière et al., 1992) respectively.

### Empathy Questionnaire; EmQue, EmQue-French-Version

The Empathy Questionnaire (Rieffe et al., 2010), translated in French by Nader-Grosbois and Simon (2019), evaluates adults' perceptions of children's empathy through 19 items. Parents estimate how frequently children's empathic reactions and/or behaviors have occurred in the last 2 months on a four-point Likert scale for each item, from “never” (1) to “always” (4). Three scores are obtained, referring to the first stages of Hoffman's developmental model (Hoffman, 2000). *Emotion contagion* (six items) refers to the intense distress felt by children when observing others' distress. *Attention for Others' Feelings* (seven items) concerns the awareness of children that, if another person feels distressed, this distress is not their own feeling. *Prosocial Actions* (six items) correspond to the capacity of children to react to others' emotions by helping, comforting, or sharing, for instance (Rieffe et al., 2010). The internal consistency of the original version of the EmQue was good for “Attention for Others' Feelings” and “Prosocial Actions,” with Cronbach's alphas of 0.71 and 0.81, respectively. This indicator was lower but still acceptable for the “Emotion Contagion” scale (0.58). In the present study, Cronbach's alphas varied between 0.68 and 0.78.

### Griffith Empathy Measure (GEM, GEM-French-Version)

This hetero-reported questionnaire (GEM, Dadds et al., 2008), adapted from the Bryant's Index of Empathy for Children and Adolescents (Bryant, 1982) and translated in French by Nader-Grosbois et al. (2019) assesses parents' perceptions of affective and cognitive empathy in their children aged between 4 and 16 years old. For each item, the parents rate children's behaviors on a nine-point Likert scale, ranging from “Strongly disagree” (−4) to “Strongly agree” (4). Of the 23 items, 6 concern cognitive empathy, 9 concern affective empathy, and 8 combine both cognitive and affective empathy, giving a total empathy score. Cronbach's alphas in the original version of this questionnaire were 0.81 for all items, 0.62 for cognitive empathy, and 0.83 for affective empathy. In the present study, Cronbach's alpha varied between 0.55 and 0.75.

### Bipolar Rating Scales Based on the Five-Factor Model (EBMCF)

This hetero-reported questionnaire (EBMCF, Roskam et al., 2000), containing 25 items, measures parents' perceptions of children's personality. For each item, parents place children on a continuum formed by a nine-point scale whose positive and negative poles consist of a pair of adjectives (e.g., shy—self-confident) reflecting opposite personality facets. Five factors are differentiated, with five items for each one: extraversion, agreeableness, conscientiousness, emotional stability, and openness. The “extraversion” factor refers to children who experience positive emotions, appreciate having contact with others, need stimulation, and are full of energy. The “agreeableness” factor concerns children who are compassionate, cooperative, helpful, and cooperative. The “conscientiousness” factor describes children who are organized, self-controlled, and efficient. The “emotional stability” factor corresponds to children who are viewed as less emotionally reactive, and stable, self-confident, and calm. Finally, the “openness” factor concerns children who are curious, creative, and open to experiences and novelty. The validation study shows that this questionnaire revealed a good internal consistency, with Cronbach's alphas of between 0.70 and 0.93. In the present study, the Cronbach's alphas varied from 0.65 to 0.86.

### Social Competence and Behavior Evaluation Scale (SCBE)

SCBE (LaFrenière et al., 1992) assesses parents' perceptions of children's social competences. Through 80 items, parents evaluate how often the child's behaviors occur, using a six-point Likert scale from “never” (0) to “always” (5). A socio-affective profile is established through eight dimensions on the basis of 10 items: angry–tolerant, anxious–secure, isolated–integrated, dependent–autonomous, resistant–cooperative, aggressive–controlled, egoistic–prosocial, and depressive–happy. For each dimension, children's weaknesses and strengths are considered on a continuum between the positive and negative poles. Some of these dimensions concern interactions with peers (isolated–integrated, egoistic–prosocial, aggressive–controlled) or adults (dependent–autonomous, resistant–cooperative), while others are related to the affective domain (depressive–happy, angry–tolerant, and anxious-secure dimensions). For each scale and the global scales, the higher the score, the less behavioral/affective difficulties the child has. Four global scales can be considered by grouping several dimensions. The externalizing problems scale includes four dimensions (angry–tolerant, resistant–cooperative, egoistic–prosocial, and aggressive–controlled), while the internalizing problems scale takes into account the other four (anxious–secure, depressive–happy, isolated–integrated, and dependent–autonomous). For these two global scales, a high score reflects the absence of internalizing or externalizing problems. The social competence scale includes affective maturity and social adjustment in interactions with peers and adults, on the basis of 40 positive items. Finally, the total score for the 80 items gives a score for general adjustment. In the present study, only social competence, externalizing, and internalizing problems were taken into account. The scores of the four scales and the eight dimensions can be converted into T-scores to compare children's skills with standard levels according to gender and age. In this way weaknesses and strengths can be identified when T-scores are lower than 38 or higher than 68, respectively. Children with a T-score between 38 and 68 thus have a non-clinical profile. Internal consistency of the French version of the SCBE is good, with Cronbach's alphas varying from 0.79 to 0.91 for the eight dimensions. The correlations for inter-judge agreement are 0.79 and 0.82, and for test–retest reliability, from 0.70 to 0.87.

### Procedure

The Hospital-Faculty Ethics Committee of Saint-Luc-UCLouvain and the Ethics Committee of the Psychological Sciences Research Institute UCLouvain approved this research procedure. Recruitment took place on a voluntary basis, through an invitation to participate being issued through kindergartens and social media. A brief document, explaining the aims of the research project and the conditions for participation, was communicated to parents who expressed an interest and a consent form was sent to them. Only questionnaires were used, as public health rules in connection with the Covid-19 pandemic ruled out the use of other performance-based measures with the children. The EBMCF and the SCBE were completed by the two parents together, while the two questionnaires on empathy, the GEM-vf and the EmQue-vf, were completed separately by both mothers and fathers. These four questionnaires were completed either on a paper version or online. At the end of their participation, parents, or children received a small gift.

## Results

### Descriptive Statistics

Table 1 presents descriptive statistics, indicating means and standard deviations for the sample's demographic and individual children's characteristics (including chronological age and personality) and empathic and social competences as perceived by parents. The EBMCF scales give indications about children's personality facets while the empathy measures provide information about children's strengths and weaknesses. However, these different scores are not compared to standards. In terms of personality, children are situated on a continuum from less to more extrovert, open to experience, emotionally stable, conscientious, and agreeable. For the empathy questionnaires, children's scores are not below average for the GEM-vf and the EmQue-vf. However, both boys and girls have higher scores on the “Attention for Others' Feelings” scale of the EmQue-vf. For the SCBE, girls obtained T-scores of 49, 49, and 40 for social competences, internalizing problems, and externalizing problems, respectively, whereas boys obtained 51, 40, and 45, respectively. For both girls and boys, the results showed that their social competences were representative of a typically developing sample, as their T-scores lay between 38 and 68. In other words, they did not have clinical internalizing and externalizing problems. Regarding their T-scores; the eight dimensions provided more details about children's socioaffective profiles. Boys and girls were less autonomous (T-score of 46 for both groups) and less cooperative in their interactions with adults (T-scores of 45 and 38, respectively), more egoistic with their peers (T-scores of 49 and 33, respectively), but more integrated (T-scores of 52 and 53, respectively). Moreover, girls were perceived as more aggressive (T-scores of 43) than boys, who were more controlled in interactions with other children (T-scores of 51). Both of these groups are perceived as joyful (T-score of 52 for boys and girls).

**Table 1:** Mean scores and standard deviations in children's characteristics, empathic skills as perceived by both mothers and fathers, and social competences as perceived by parents.

**Children's variables**	**Girls**	**Boys**	**Total**
	***M* (SD)**	***M* (SD)**	***M* (SD)**
**Children's characteristics**			
Sample	36	28	64
Age (in months)	53.89 (10.19)	55.59 (12.49)	54.62 (11.17)
Extraversion (max = 9)	6.80 (1.28)	6.50 (1.24)	6.65 (1.26)
Emotional stability (max = 9)	4.96 (1.15)	5.40 (1.26)	5.14 (1.21)
Conscientiousness (max = 9)	6.40 (1.33)	6.27 (1.17)	6.34 (1.26)
Openness (max = 9)	7.82 (0.77)	7.88 (0.84)	7.84 (0.79)
Agreeableness (max = 9)	6.80 (0.98)	7.08 (0.78)	6.91 (0.91)
**Children's empathic skills**			
**Perceived by mothers**			
Emotion contagion (max = 24)	11.00 (2.95)	10.16 (2.59)	10.63 (2.80)
Attention for others' feeling (max = 28)	20.40 (4.24)	20.52 (3.02)	20.45 (3.73)
Prosocial actions (max = 24)	13.61 (2.56)	13.72 (3.28)	13.66 (2.88)
Affective empathy (max = 4)	0.51 (1.02)	0.33 (1.10)	0.43 (1.05)
Cognitive empathy (max = 4)	0.54 (1.16)	1.17 (1.21)	0.71 (1.28)
Empathy total (max = 4)	1.17 (0.85)	1.09 (0.77)	1.14 (0.81)
**Children's empathic skills**			
**Perceived by fathers**			
Emotion contagion (max = 24)	11.77 (2.75)	10.88 (2.11)	11.42 (2.52)
Attention for others' feeling (max = 28)	20.84 (2.97)	19.22 (2.73)	20.18 (2.95)
Prosocial actions (max = 24)	13.48 (2.27)	14.33 (2.58)	13.82 (2.41)
Affective empathy (max = 4)	0.60 (0.71)	0.18 (1.01)	0.44 (0.85)
Cognitive empathy (max = 4)	0.71 (1.28)	0.71 (1.47)	0.71 (1.34)
Empathy total (max = 4)	1.16 (0.88)	0.79 (0.74)	1.02 (0.84)
**Children's social adjustment**			
Social competences (max = 200)	130.89 (17.81)	128.04 (22.06)	129.67 (19.63)
Internalizing problems (max = 100)	74.20 (10.00)	71.88 (13.79)	73.21 (11.73)
Externalizing problems (max = 100)	63.49 (11.78)	65.54 (13.51)	64.37 (12.48)
Depressed–joyful (max = 50)	39.77 (4.12)	39.82 (5.49)	39.79 (4.71)
Anxious–confident (max = 50)	37.65 (5.46)	36.48 (5.29)	37.15 (5.38)
Angry–tolerant (max = 50)	24.97 (5.86)	27.48 (6.27)	26.04 (6.12)
Isolated–integrated (max = 50)	39.43 (4.71)	37.26 (6.99)	38.51 (5.84)
Aggressive-controlled (max = 50)	34.95 (4.36)	33.94 (4.64)	34.52 (4.47)
Egoistic–prosocial (max= 50)	28.42 (6.16)	28.80 (5.26)	28.59 (5.75)
Resistant-cooperative (max = 50)	33.26 (5.43)	34.19 (6.17)	33.65 (5.72)
Dependent–autonomous (max = 50)	31.98 (6.84)	32.38 (5.32)	32.15 (6.20)

### Mothers' and Fathers' Perceptions of Children's Empathy

To investigate the differences between mothers' and fathers' perception of children's empathy, several paired sample *t*-tests were used. Only one significant difference between mothers and fathers was obtained, concerning children's emotion contagion (*t* = −2.092, *p* = 0.042). Fathers perceived their children as being more overwhelmed by others' emotions than mothers (see Table 2 for means and standard deviations). Analyses brought out no other significant difference between parents' perception of children's empathy, in scores for other scales of the EmQue-vf and the GEM-vf.

**Table 2:** Means, standard deviations, and *t*-test concerning parents' perception of children's empathy.

**Empathy variables**	**Mothers**	**Fathers**		
	***M* (SD)**	***M* (SD)**	***t***	***d***
Affective empathy	0.43 (1.05)	0.44 (0.85)	0.882	0.01
Cognitive empathy	0.71 (1.28)	0.71 (1.34)	0.492	0.00
Empathy total	1.14 (0.81)	1.02 (0.84)	1.669	0.14
Emotion contagion	10.63 (2.80)	11.42 (2.52)	−2.092*	0.29
Attention for others' feelings	20.45 (3.73)	20.18 (2.95)	0.509	0.08
Prosocial actions	13.66 (2.88)	13.82 (2.41)	−0.349	0.06

* *p < 0.05*.

### Children's Empathy in Link With Gender, Age, and Personality

Before running analyses, it was considered to aggregate mothers' and fathers' scores. Therefore, a factorial analysis has been realized to test if those scores of affective and cognitive empathy obtained by mothers and fathers loaded on the same factors. As it was not the case, separated scores has been kept.

To examine potential gender differences in children's empathy, two separate one-way MANOVA analyses were conducted, for maternal reports and paternal reports. For maternal perceptions of children's empathic skills, the one-way MANOVA showed no effect of gender (*F* = 1.562; *p* = 0.188; η^2^ = 0.135). However, tests of between-subjects demonstrated a significant difference depending on gender in cognitive empathy (*F* = 9.525; *p* = 0.013; η^2^ = 0.109), in the sense that boys scoring higher than girls (see Table 1 for means). No gender difference was obtained by the one-way MANOVA of paternal perceptions of children's empathy (*F* = 2.467; *p* = 0.051; η^2^ = 0.261). On the scale of attention for others' feelings, a significant gender difference was identified by the test of between-subjects (*F* = 5.844; *p* = 0.020; η^2^ = 0.130), indicating that girls were perceived by their fathers as more attentive to others' feelings than boys (see Table 1 for means).

Table 3 presents the intercorrelations between children's empathic abilities and their individual characteristics (age and personality). It reveals that age is only positively related to the total score of the GEM completed by mothers (*r* =*0.3*45; *p* = 0.006). Concerning personality factors, extraversion (*r* = −0.538; *p* = 0.000), emotional stability (*r* = 0.318; *p* = 0.034), and agreeableness (*r* = −0.331; *p* = 0.026) were negatively correlated with emotion contagion as rated by fathers, while emotional stability (*r* = −0.296; *p* = 0.021) was negatively linked to affective empathy as perceived by mothers. Openness to experience and agreeableness was positively and significantly related to cognitive empathy as rated by mothers (*r* = 0.297; *p* = 0.020; *r* = 0.443; *p* = 0.000, respectively) and to prosocial actions as rated by both mothers (*r* = 0.298; *p* = 0.027; *r* = 0.342; *p* = 0.011, respectively) and fathers (*r* = 0.357; *p* = 0.016; *r* = 0.367; *p* = 0.013, respectively).

**Table 3:** Spearman correlations between children's individual characteristics and skills in empathy rating by mothers and fathers.

**Children's characteristics**	**Age**	**Extraversion**	**Stability**	**Conscientiousness**	**Openness**	**Agreeableness**
**Empathy variables**						
**Perceived by mothers**						
Emotion contagion	−0.085	−0.137	−0.191	−0.090	0.107	0.006
Attention for others' feelings	−0.109	0.009	−0.124	0.111	0.244	0.180
Prosocial actions	0.049	0.009	−0.030	0.154	0.298*	0.342*
Affective empathy	0.105	−0.044	−0.296*	−0.057	0.007	0.101
Cognitive empathy	0.103	0.213	0.123	0.076	0.297*	0.443***
Empathy total	0.345**	0.054	−0.078	0.093	0.170	0.191
**Perceived by fathers**						
Emotion contagion	−0.273	−0.538***	−0.318*	−0.156	−0.053	−0.331*
Attention for others' feelings	−0.260	0.034	−0.013	0.132	−0.024	−0.064
Prosocial actions	0.085	0.147	−0.120	0.019	0.357*	0.367*
Affective empathy	−0.140	−0.30	−0.283	0.072	−0.110	−0.138
Cognitive empathy	−0.100	−0.049	0.035	0.121	0.126	0.259
Empathy total	0.176	0.240	−0.177	0.170	0.142	0.141

* *p < 0.05*;

** *p < 0.01*;

*** *p < 0.001*.

Several multiple linear regressions with a stepwise method were performed to explore the extent to which children's age and personality could predict their empathic abilities. Children's ages were entered in Step 1 and the five factors of children's personality in Step 2. The variance inflation index (VIF) was used to control multicollinearity. There was no multicollinearity between variables in the different analyses. Tables 4, 5 present the results for significant predictors of children's empathy as perceived, respectively, by their mothers (Table 4) and their fathers (Table 5). Emotional stability explained 6.8 and 7.8% of the variance in affective empathy scores given by mothers and fathers, respectively, while agreeableness explained 14.3% of the variance in children's cognitive empathy as evaluated only by mothers. Regarding the total score for the GEM-vf, 6.4% of the variance was explained by age when empathy was rated by mothers. Agreeableness explained 10.7% of the variance in prosocial actions in the EmQue-vf, rated by mothers, while openness to experience explained 8.5% of the variance of prosocial actions as assessed by fathers. Model 3f, including age (β = −0.097, *p* < 0.494), extraversion (β = −0.430, *p* < 0.004), and emotional stability (β = −0.295, *p* < 0.027), explained 27.9% of the variance in emotion contagion as rated by fathers. Before personality was integrated in the model, age remained a significant predictor and explained 6.9% of the variance in emotion contagion.

**Table 4:** Predictors of children's empathy skills as perceived by Mothers according to children's individual characteristics.

**Predictors**	***B***	***SE/B***	**β**	$R_{\mathrm{adj}}^{2}$	***F***
**Affective empathy**					
Model 1a				0.068	5.391*
Stability	−0.252	0.109	−0.289*		
**Cognitive empathy**					
Model 1b				0.143	11.014**
Agreeableness	0.528	0.159	0.397**		
**Empathy total**					
Model 1c				0.064	5.136*
Age	0.021	0.009	0.283*		
**Prosocial actions**					
Model 1d				0.107	7.474**
Agreeableness	1.124	0.411	0.352**		

* *p < 0.05*;

** *p < 0.01*.

**Table 5:** Predictors of children's empathy skills as perceived by fathers according to children's individual characteristics.

**Predictors**	***B***	***SE/B***	**β**	$R_{\mathrm{adj}}^{2}$	***F***
**Affective empathy**					
Model 1e				0.078	4.468*
Stabillity	−0.217	0.103	−0.317*		
**Emotion-contagion**					
Model 1f				0.069	4.251*
Age	−0.071	0.034	−0.300*		
Model 2f				0.206	6.708**
Age	−0.030	0.035	−0.129		
Extraversion	−0.861	0.297	−0.426**		
Model 3f				0.279	6.668***
Age	−0.23	0.033	−0.097		
Extraversion	−0.869	0.283	−0.430**		
Stability	−0.601	0.263	−0.295*		
**Prosocial actions**					
Model 1g				0.085	5.089*
Openness	0.933	0.413	0.325*		

* *p < 0.05*;

** *p < 0.01*;

*** *p < 0.001*.

### Children's Social Adjustment in Link With Their Personality Factors and Empathy

Table 6 presents the intercorrelations between children's social competences and internalizing and externalizing problems in SCBE on the one hand and their personality and empathy on the other. Social competences are positively and significantly linked with factors of personality, except that no such link was found between emotional stability (*r* between 0.255 and 0.490; *p* between 0.000 and 0.047) and attention for others' feelings as rated by mothers (*r* = 0.413; *p* = 0.001), cognitive empathy as rated by mothers (*r* = 0.395; *p* = 0.001) and by fathers (*r* = 0.372; *p* = 0.015), or the total GEM score as assessed by fathers (*r* = 0.315; *p* = 0.042). Concerning internalizing problems, positive correlations were found with all factors of personality, except for conscientiousness and openness to experience (*r* between 0.295 and 0.438; *p* between 0.000 and 0.021); this positive correlation was also found with cognitive empathy only as rated by mothers (*r* = 0.399, *p* = 0.001). Externalizing problems were positively and significantly related to emotional stability (*r* = 0.485, *p* = 0.000), agreeableness (*r* = 0.377, *p* = 0.003), and cognitive empathy as assessed by mothers (*r* = 0.355; *p* = 0.004) and fathers (*r* = 0.326, *p* = 0.035), and negatively correlated with affective empathy as perceived by mothers (*r* = −0.294, *p* = 0.019) and fathers (*r* = −0.316, *p* = 0.041).

**Table 6:** Spearman correlations between children's individual characteristics, empathy and social skills, and behavioral problems.

	**Social competences**	**Internalizing problems**	**Externalizing problems**
Age	0.012	0.193	0.032
Extraversion	0.255*	0.438**	−0.003
Stability	0.070	0.359**	0.485***
Conscientiousness	0.266*	0.212	0.222
Openness	0.471***	0.266*	0.471***
Agreeableness	0.490***	0.295*	0.377**
**Empathy as perceived by mothers**			
Emotion contagion	0.168	−0.194	−0.131
Attention for others' feelings	0.413***	0.041	−0.057
Prosocial actions	0.444**	0.131	0.218
Affective empathy	−0.008	−0.246	−0.294*
Cognitive empathy	3.95***	0.399***	0.355**
Empathy total	0.194	−0.073	−0.072
**Empathy as perceived by fathers**			
Emotion contagion	−0.180	−0.285	−0.159
Attention for others' feelings	0.133	0.173	−0.058
Prosocial actions	0.408**	0.202	0.202
Affective empathy	−0.178	−0.302	−0.316*
Cognitive empathy	0.372*	0.161	0.326*
Empathy total	0.315*	−0.095	−0.060

* *p < 0.05*;

** *p < 0.01*;

*** *p < 0.001*.

Linear regression analyses using a stepwise method were performed to explore the part of variance of social adjustment explained by empathy and personality. The focus was on the extent to which children's empathy and personality predicted the variance in the three global scores of the SCBE, including social competences, internalizing problems, and externalizing problems. Two separate models were presented, the first incorporating empathic skills as rated by mothers and the second as rated by fathers. Children's ages were entered in Step 1, the five personality factors of the EBMCF in Step 2, and the affective and cognitive scales from the GEM-vf and the emotion contagion and attention for others' feelings scales from the EmQue-vf in Step 3. The prosocial actions scale of the EmQue was not entered because this scale evaluated social behaviors too similar to those included in the dependent variable measure. Multicollinearity was controlled for and values did not exceed 1. There was no multicollinearity between variables.

Table 7 presents the results for significant predictors of children's social adjustment, depending on children's individual characteristics and their empathic abilities as perceived by mothers. Model M3a, including openness (β = 0.359, *p* < 0.003), agreeableness (β = 0.255, *p* < 0.034), and attention for others' feelings (β = 0.257, *p* < 0.026), explained 36.2% of the variance in social competences. In Model M3b, 22.8% of the variance in internalizing problems was explained by extraversion (β = 0.253, *p* < 0.042), emotional stability (β = 0.259, *p* < 0.034), and cognitive empathy (β = 0.316, *p* < 0.012). Model M2c, with personality as predictor, explained 22.6% of the variance in externalizing problems: significant predictors were emotional stability (β = 0.334, *p* < 0.008) and agreeableness (β = 0.317, *p* < 0.012).

**Table 7:** Predictors of children's social skills according to children's individual characteristics and their skills in empathy as perceived by mothers.

**Predictors**	***B***	***SE/B***	**β**	$R_{\mathrm{adj}}^{2}$	***F***
**Social competences**					
Model M1a				0.245	18.814***
Openness	12.665	2.920	0.508***		
Model M2a				0.311	13.397***
Openness	9.928	2.999	0.398**		
Agreeableness	6.918	2.783	0.299*		
Model M3a				0.362	11.407***
Openness	8.938	2.917	0.359**		
Agreeableness	5.909	2.714	0.255*		
Attention for others' feelings	1.414	0.616	0.257*		
**Internalizing problems**					
Model M1b				0.076	5.504*
Extraversion	3.153	1.344	0.304*		
Model M2b				0.144	5.624**
Extraversion	3.307	1.295	0.319*		
Stability	2.723	1.185	0.28*		
Model M3b				0.228	6.400***
Extraversion	2.618	1.258	0.253		
Stability	2.454	1.128	0.259		
Cognitive empathy	2.949	1.136	0.316*		
**Externalizing problems**					
Model M1c				0.143	10.146**
Stability	4.000	1.256	0.398**		
Model M2c				0.226	9.044***
Stability	3.358	1.218	0.334*		
Agreeableness	4.510	1.724	0.317*		

* *p < 0.05*;

** *p < 0.01*;

*** *p < 0.001*.

Table 8 presents the significant predictors of children's social adjustment, depending on children's empathic skills and their personality as perceived by fathers. Model F1a, including openness (β = 0.532, *p* < 0.000) and cognitive empathy (β = 0.281, *p* < 0.033), explained 36.7% of the variance in social competences. Model F3b, composed of emotional stability (β = 0.388, *p* < 0.003), extraversion (β = 0.445, *p* < 0.001), and cognitive empathy (β = 0.371, *p* < 0.005), explained 40.2% of the variance in internalizing problems. Model F2c, comprising emotional stability (β = 0.411, *p* < 0.006) and cognitive empathy (β = 0.294, *p* < 0.042), explained 22.2% of the variance in externalizing problems.

**Table 8:** Predictors of children's social skills according to children's individual characteristics and their skills in empathy as perceived by fathers.

**Predictors**	***B***	***SE/B***	**β**	$R_{\mathrm{adj}}^{2}$	***F***
**Social competences**					
Model F1a				0.303	18.393***
Openness	12.346	2.879	0.566***		
Model F1a				0.367	12.585***
Openness	11.602	2.764	0.532***		
Cognitive empathy	3.968	1.788	0.281*		
**Internalizing problems**					
Model F1b				0.137	7.355**
Stability	3.709	1.368	0.422*		
Model F2b				0.276	8.643***
Stability	3.707	1.252	0.433**		
Extraversion	3.577	1.226	0.355*		
Model F3b				0.402	9.973***
Stability	3.613	1.139	0.388**		
Extraversion	4.059	1.126	0.445***		
Cognitive empathy	3.262	1.088	0.371**		
**Externalizing problems**					
Model F1c				0.154	8.280**
Stability	3.981	1.383	0.418**		
Model F2c				0.222	6.723**
Stability	3.905	1.327	0.411**		
Cognitive empathy	2.641	1.254	0.294*		

* *p < 0.05*;

** *p < 0.01*;

*** *p < 0.001*.

## Discussion

The aims of this study were to investigate how mothers and fathers perceive their child's empathic abilities according to developmental stages and affective and cognitive components (1), in order to study whether their perceptions of empathy vary depending on children's individual factors, including gender, age, and personality (2), and to examine how social competences or internalizing and externalizing behaviors are linked with children's cognitive and affective empathy and personality (3).

No previous study has compared mothers' and fathers' perceptions of affective and cognitive empathy in children at preschool age. As it was hypothesized, the results of this study showed similar levels in both cognitive and affective components of empathy, in “attention to others' feelings” and in “prosocial actions.” As fathers are spending more and more time with their children (Smeaton and Marsh, 2006), it was expected that they would be found to observe their children's behaviors in interactions with peers or adults in the same way as mothers. Moreover, it was hypothesized that parents share common values about the importance of interest in others, empathy, and prosociality in their children's upbringing. However, the results indicated one significant difference, in that fathers were more inclined than mothers to perceive their child as displaying emotion contagion. This perhaps suggests that these fathers' tolerance of the emotion contagion or regulation displayed by their child in social critical situations differed from that of the mothers. Because fathers would like that their child becomes more independent, they could pay more attention to the way their child controls his or her emotions. Therefore, small emotional reactions by a child to others' feelings could be considered as emotion contagion by their father. Conversely, mothers could see their child as more competent to regulate his or her emotions and less overwhelmed by other's emotions.

In terms of gender similarities or differences, the comparison of girls' and boys' empathic abilities revealed that fathers perceived girls as paying more attention to others' feelings than boys, i.e., as displaying more affective empathy. This result was largely consistent with the existing literature (Zahn-Waxler et al., 1992; de Wied et al., 2007; Auyeung et al., 2009; Lucas-Molina et al., 2018). As argued by Strauss (2004), in our society children assume behaviors and attitudes which correspond to their gender at an early age. Therefore, girls display more affective empathy and behave more prosocially than boys. This difference between girls and boys implicitly reflects behaviors and attitudes may be valorized differently with respect of their social roles. That correspond to their future caregiver roles. Concerning emotion contagion and affective empathy as perceived by mothers or fathers, no difference according to gender was highlighted in our results, in line with Bensalah et al. (2016) and Schwenck et al. (2014). However, boys were perceived by their mothers as having better cognitive empathy than girls. Our results corroborated those of Volbrecht et al. (2007), who found in an observational measure that boys aged between 12 and 25 months engaged more in hypothesis testing (considered as an indicator of cognitive empathy at an early age) to understand their mother's distress than girls. However, even if the present results corroborate with those of some studies, it seems to be difficult to reach out a general conclusion about the gender factor in the development of children's empathy. Indeed, as reported by Fabes and Eisenberg (1998), studies used different kinds of measures to assess empathic abilities and concerned different age ranges.

As expected in the second hypothesis, the linear regressions showed that personality factors, and more precisely emotional stability, extraversion, agreeableness, and openness to experience, are associated with empathy. Concerning the affective component, emotional stability was negatively related to the affective empathy score of the GEM-vf, as evaluated by mothers and fathers. These results are coherent with those of Jolliffe and Farrington (2006a) who found that neuroticism, the reverse of emotional stability, is positively correlated with affective empathy in adolescent girls, indicating that certain facets of neuroticism (like self-consciousness and guilt) might ease the empathic experience. Moreover, Cornell and Frick (2007) obtained the same results for preschool age by studying the link between inhibited temperament, characterized by anxiety and fearfulness, and global empathy. Children with an inhibited temperament, characterized as shy and fearful with unknown people or situations, are characterized as more empathic by parents. These results support the claim of Blair (1999) and Kochanska (1993) that children with some difficulties in inhibiting their behaviors lack what is regarded as a precursor of empathy: they are not affected by others' distress. Similarly, our results showed that extraversion and emotional stability are negatively related to emotion contagion as perceived by fathers. In other words, children who experience positive emotions are calmer and more stable, and children who are less emotionally reactive are less affected by others' emotions, as was reported by Jolliffe and Farrington (2006a) and Liew et al. (2011). In accordance with the definition of agreeableness (Mervielde and De Fruyt, 1999), our results showed that agreeable children are perceived by their mothers as having more cognitive empathy. Agreeableness is characterized by compassion, cooperation, consideration, help and seeking social harmony. Conversely, disagreeable children do not generally take an interest in others' well-being and may appear cold toward others (Mervielde and De Fruyt, 1999). Conceivably, children's desire to help others implies that their understanding of what they feel leads them to adapt their behavior to their needs. Children's prosocial actions as perceived by mothers in EmQue-vf are positively related to agreeableness, while the same component as perceived by fathers is positively linked to openness to experience. These two personality factors are also positively related to prosocial behaviors in the results obtained by Tariq and Naqvi (2020). Moreover, it makes sense that agreeableness and prosocial behaviors are associated, in view of the definition of this personality factor (Mervielde and De Fruyt, 1999). Concerning openness to experience, children with a high level of this personality factor are more curious, creative, and imaginative, while children who are less open to experience resist change and prefer proximity and familiarity (Mervielde and De Fruyt, 1999). Less open children may also be less likely to help other children in distress, as they may be more resistant to other's perspectives and emotions.

Age was only positively related to the total score of the GEM-vf, in line with the developmental model of empathy of Hoffman (2000). However, the specific affective and cognitive components of empathy were not significantly linked to age in these preschool children, contrary to the results reported by Bensalah et al. (2016) and Schwenck et al. (2014). Moreover, emotion contagion, as perceived by fathers, decreased as children grow up, as hypothesized by Rieffe et al. (2010). However, when personality factors were added as predictor variables in the analyses, age ceased to be a significant predictor. It seems that personality explains a greater part of the variance in empathy than age, at preschool level. Therefore, the second hypothesis concerning the influence of age on empathic skills is partially confirmed.

Numerous studies have investigated the relation between empathy and social competences or externalizing behaviors, but few have analyzed the link between empathy and internalizing behaviors. In the present study, it was hypothesized that empathy perceived by mothers and fathers, as well as personality factors (emotional stability, agreeableness, extraversion, openness to experience) could be positively related to children's social competences. Moreover, the third hypothesis suggests that empathy and those personality factors could contribute to be protective factors against internalizing and externalizing problems. Concerning social competences, it was observed that attention to others' feeling as perceived by mothers, openness to experience and agreeableness are positively related to social competences. In other words, open and agreeable children who pay more attention to others' emotions and distress are more socially adjusted in interactions with peers or adults; this is consistent with the definitions of openness and agreeableness (Mervielde and De Fruyt, 1999) and reflects the results of affective empathy observations (Belacchi and Farina, 2012; Girard et al., 2014; Hirn et al., 2019). The significant predictive value of openness to experience for social competences was revealed when paternal perceptions of empathy were considered. Indeed, children who were perceived by their fathers as more competent in cognitive empathy had better social competences; this corroborates the results reported by Hirn et al. (2019) on adolescents. These results suggested that children's affective and cognitive empathy, as well as their openness to experience and their agreeableness are positively related to their social competences. Therefore, it could be considered that empathy and personality are favorable factors in social competences development at preschool age.

Little previous research has been conducted about the relation between empathy and internalizing behaviors; our study demonstrated that emotional stability, extraversion, and cognitive empathy, as perceived by mothers and by fathers, are negatively related to internalizing behaviors. In other words, stable and extrovert children with better cognitive empathy are less likely to display internalizing behaviors at preschool age. Children with stable and extrovert personalities are defined as presenting most social characteristics and as being little affected by others' emotions (Mervielde and De Fruyt, 1999); this is thought to play a role in protecting them against developing internalizing behaviors (Slobodskaya and Akhmetova, 2010; Delgado et al., 2018). Conversely, more introvert and neurotic children are more anxious, withdrawn, and disengaged in social activities, which is similar to what is observed in children with internalizing problems (Achenbach et al., 1987). As predicted by Raine and Chen (2018), our results showed that high cognitive empathy abilities reduced the risk of internalizing problems. However, according to Tone and Tully (2014), when children display an extreme level of empathy, this may contribute to a higher level of internalizing behaviors, particularly when other risk factors in the family (e.g., mother's depression) are involved. Although our sample consisted entirely of typically developing children without pathological internalizing disorders, it should be stressed that both affective and cognitive empathy and personality factors could be considered as protective factors against the development of internalizing behaviors.

The multiple linear regressions found that, when maternal perceptions of affective and cognitive empathy was considered, only personality was negatively related to externalizing problems, characterized by impulsivity, aggressivity, opposition, or disobedience (Achenbach et al., 1987). In other words, agreeable and stable children, who are friendly, generous, helpful, calm, and less emotionally reactive (Mervielde and De Fruyt, 1999), present fewer externalizing behaviors, as found by Meunier et al. (2011). Empathy as rated by mothers did not appear as a significant predictor of externalizing problems. By contrast, cognitive empathy as perceived by fathers and emotional stability did predict a lower level of externalizing behaviors. This result is consistent with the third hypothesis of this study and the findings reported by Belacchi and Farina (2012), that children perceived as more hostile have lower levels of cognitive empathy. However, some adolescents who bully others may present high cognitive empathy, as observed by Jolliffe and Farrington (2006b). The same conclusion has been reported by Jones et al. (2010) in the case of children and adolescents with psychopathic traits who present antisocial behaviors. Regarding these controversial results, it is possible that empathy and personality have been considered as protective factors for externalizing behaviors in children except for those who present psychological disorders. Further investigations are therefore needed to throw more light on this relation.

This exploratory research makes new contributions to the field of empathy and socio-emotional development at preschool age. It shows the relevance of taking account of the respective influence of personality, gender, and the multi-dimensional aspects of empathy in order to gain a clearer picture of how empathy profiles could be linked to social competences vs. maladjustment risk. Looking at the perceptions of fathers and mothers concerning their children's empathy profiles offers new ways of understanding their socio-emotional development. For research and prevention purposes, multi-informant assessment may help to detect strengths or weaknesses in empathy profiles and social adjustment in children. Future research could test whether specific prevention or interventions in classroom targeting SIP or theory of mind and adapted to preschool age (e.g., such as ToM and SIP program conceived by Honoré et al., 2020) improve empathy profiles beyond social adjustment.

Although this study helps to refine knowledge about the development of social and emotional skills, some limitations should be taken in account. First, the sample size of 63 typically developing children, and especially when the sample is divided into boys and girls, is small and the questions need to be investigated in a greater sample in families from more diverse socio-cultural and socioeconomic backgrounds. Second, this study used only other-reported questionnaires and not performance-based measures of empathy. It may be interesting to involve children in the data collection with observational settings or by asking them questions about stories. In another study in the research project, observational and performance-based measures and questionnaires about empathy have been used in order to examine these questions in greater depth. Asking teachers to complete questionnaires on empathy and social competences may reveal how children display these skills in the social environment of kindergarten, in classroom, and at break. Third, it is possible that parents have some difficulties in completing some of the items of empathy questionnaires, because they have variable and limited opportunities to observe their children in interaction with others in critical situations or in distress. The final limitation concerns the use of measures of empathy that do not allow the level of empathy to be specified. Regarding the literature about empathy in children and adolescents with internalizing behaviors, establishing if their level of empathy is excessive or defective could refine our knowledge in this field. Unfortunately, no existing questionnaires make it possible to identify a pathological degree of empathy.

## Data Availability Statement

The raw data supporting the conclusions of this article will be made available by the authors, without undue reservation.

## Ethics Statement

The studies involving human participants were reviewed and approved by Hospital-Faculty Ethics Committee of Saint-Luc-UCLouvain and Ethics Committee of the Psychological Sciences Research Institute in UCLouvain. Written informed consent to participate in this study was provided by the participants' legal guardian/next of kin.

## Author Contributions

PS collected and analysed the data and wrote the manuscript. NN-G supervised the research and contributed to write the manuscript. Both authors approved the submitted version.

## Funding

This publication is supported by Chair Baron Frère in special education, directed by Professor Nathalie Nader-Grosbois.

## Conflict of Interest

The authors declare that the research was conducted in the absence of any commercial or financial relationships that could be construed as a potential conflict of interest.

## Publisher's Note

All claims expressed in this article are solely those of the authors and do not necessarily represent those of their affiliated organizations, or those of the publisher, the editors and the reviewers. Any product that may be evaluated in this article, or claim that may be made by its manufacturer, is not guaranteed or endorsed by the publisher.
